# Bilateral Giant Ovarian Masses in a Pre-pubertal Girl: A Case of Mature Teratoma and Torsion Presenting With Acute Urinary Retention

**DOI:** 10.7759/cureus.100307

**Published:** 2025-12-29

**Authors:** Ayodeji O Oyeniran, Kehinde Awodele, Sunday C Adeyemo, Olusegun S Oyelami, Olugbenga P Akintunde, Samuel O Omopariola, Babalola O Emmanuel, Adeniyi O Fasanu, Eniola D Olabode

**Affiliations:** 1 Department of Obstetrics and Gynaecology, Uniosun Teaching Hospital, Osun State University, Osogbo, NGA; 2 Department of Obstetrics and Gynaecology, Osun State University, Osogbo, NGA; 3 Department of Public Health Research, University of Wolverhampton, Wolverhampton, GBR; 4 Department of Health and Biomedical Sciences, Institut Superieur de Sante, Niamey, NER; 5 Department of Obstetrics and Gynaecology, All Women's Care Fertility and Specialist Hospital, Osogbo, NGA; 6 Department of Obstetrics and Gynaecology, Obafemi Awolowo Teaching Hospital Complex, Ile-Ife, NGA; 7 Department of Obstetrics, Gynaecology and Perinatology, Obafemi Awolowo University, Ile-Ife, NGA; 8 Department of Community Medicine, Institut Superieur de Sante, Niamey, NER

**Keywords:** cystectomy, laparotomy, pre-pubertal, teratoma, torsion, urinary retention

## Abstract

Mature cystic teratoma (MCT) is a common benign ovarian neoplasm, but its presentation as bilateral, giant masses in a prepubertal child is rare. Complications like torsion and acute urinary retention pose significant diagnostic and management challenges, where fertility preservation is a primary concern.

An 8-year-old premenarchal girl presented with a four-day history of abdominal pain, fever, and acute urinary retention. Examination revealed a large, firm abdominal mass. Imaging suggested bilateral immature teratoma. Emergency laparotomy revealed two massive ovarian masses, with the left torsed, ischemic, and a rudimentary uterus. Bilateral ovarian cystectomy was successfully performed. Histopathology confirmed a right-sided mature cystic teratoma and left-sided hemorrhagic necrosis from torsion, ruling out malignancy. The patient had an uneventful recovery and was commenced on hormone replacement therapy due to compromised ovarian reserve. At two-year follow-up, she remains stable with no complaints.

This case underscores that giant ovarian teratomas, though rare in children, can present dramatically. It highlights the critical importance of a fertility-preserving surgical approach even in complex cases and the necessity of long-term, multidisciplinary follow-up to manage subsequent endocrine sequelae.

## Introduction

Teratoma is a benign germ cell tumor composed of multiple cell types, originating from more than one germ layer, with the ovary being one of its most common locations [1]. Mature cystic teratomas (MCTs) of the ovary are the most common benign ovarian neoplasms in the reproductive age group, accounting for 10-20% of all ovarian neoplasms [2]. Clinically, they can be asymptomatic or present with abdominal complaints when they are large. Acute abdominal pain occurs in 5-10% of all mature teratomas and is often due to ovarian torsion, which represents a serious complication [3]. While most MCTs are unilateral, bilateral presentation is less common, representing approximately 10-15% of cases [4].

Imaging remains the cornerstone for diagnosing ovarian mature teratomas, with ultrasonography serving as the first-line modality for initial evaluation [4]. The definitive confirmation of the diagnosis is made by histopathological examination [2]. The management of these tumors is primarily surgical, and the presence of torsion constitutes a surgical emergency [3]. The surgical approach for bilateral teratomas can be challenging due to their relative uncommonness and the lack of a universal protocol, requiring careful consideration of fertility preservation and the risk of iatrogenic menopause [5].

## Case presentation

An 8-year-old pre-menarchal girl presented to the emergency unit with a four-day history of colicky lower abdominal pain, fever, and a one-day history of acute urinary retention. There was associated abdominal distension and vomiting. There is no family history of gynaecological malignancy. On examination, she was afebrile, and her vital signs were within normal limits. Her height was 1.28 m (50th percentile), weight 26 kg (50th percentile), and body mass index (BMI) 15.9 kg/m² (50th percentile), consistent with normal prepubertal growth parameters. Abdominal examination revealed significant distension with a firm, tender, non-mobile mass extending above the umbilicus. Pelvic examination showed normal infantile genitalia.

**Figure 1:**
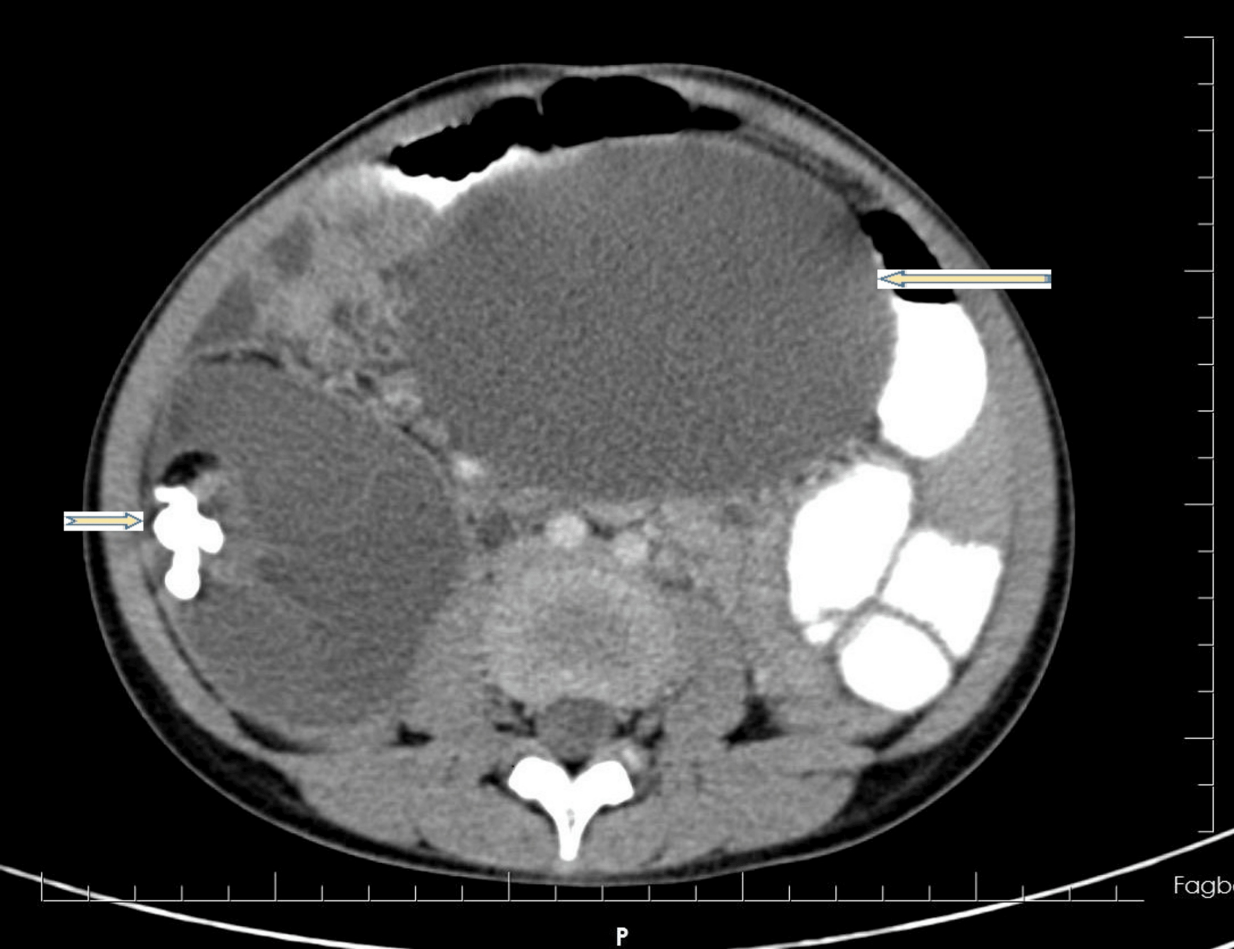
Axial contrast-enhanced CT scan of the abdomen. The image shows bilateral giant ovarian masses occupying the pelvic and lower abdominal cavities. The right-sided mass contains areas of fat and calcification (arrow), characteristic of a mature cystic teratoma. The left adnexal mass demonstrates heterogeneous density with surrounding fluid and mass effect (arrow), suggestive of ovarian torsion. The enlarged masses exert significant compressive effect on adjacent pelvic organs, correlating with the patient’s presentation of acute urinary retention.

A diagnosis of suspected ovarian malignancy, complicated by torsion and acute urinary retention, was made. An abdominopelvic ultrasound revealed complex intra-abdominal cystic masses, suspicious for an ovarian lesion. A subsequent computed tomography (CT) scan suggested bilateral immature ovarian teratoma (Figure 1).

**Figure 2:**
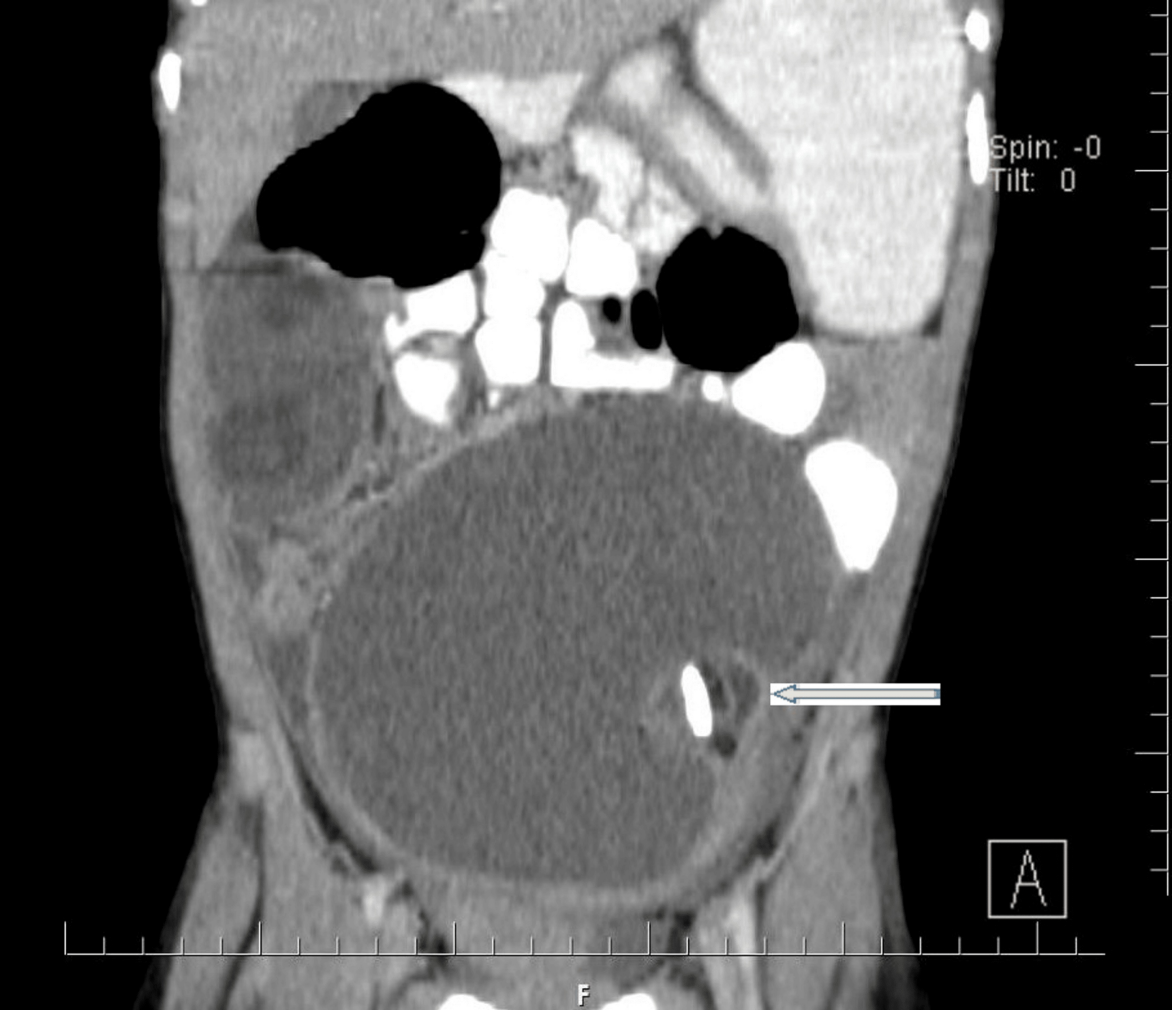
Image showing left ovarian mass.

**Figure 3:**
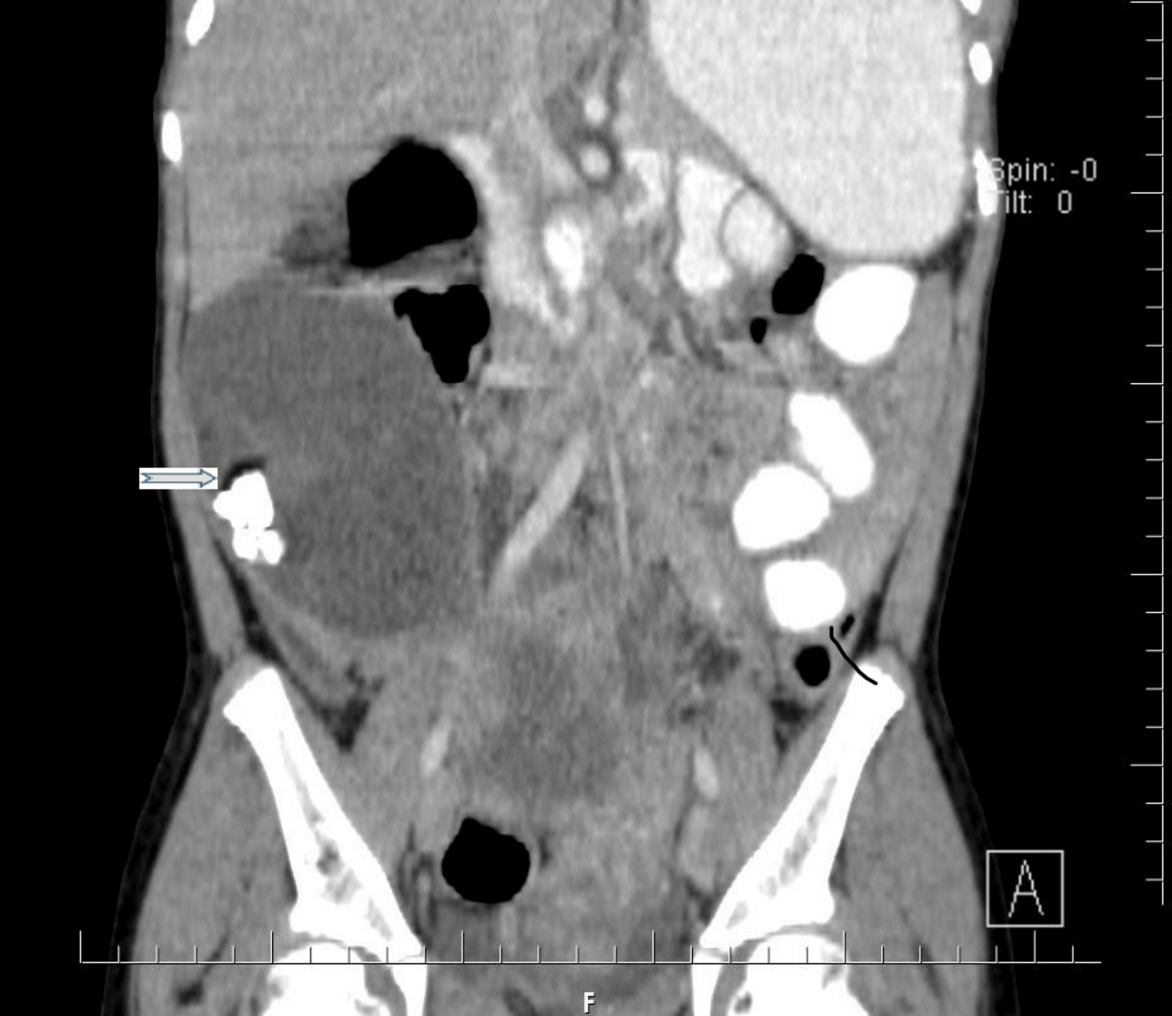
Image showing right ovarian mass.

Tumor marker CA-125 was within normal limits at 6.3 U/L. Notably, germ cell tumor markers were also assessed: alpha-fetoprotein (AFP) was 0.5 ng/mL (within normal limits), and beta-human chorionic gonadotropin (β-hCG) was 0.02 MIU/mL (within normal limits). After extensive counselling and preparation, the patient was admitted on 23rd July, 2023, and underwent an emergency exploratory laparotomy. CT findings included two huge ovarian masses: an 18x14 cm torsed and ischemic left ovarian mass (Figure 2), and a 15x14 cm right ovarian mass (Figure 3). The uterus was rudimentary. There was no evidence of peritoneal seeding. A bilateral ovarian cystectomy was successfully performed on 28th July, 2023.

Gross examination

The excised bilateral ovarian masses were submitted for gross and microscopic examination. Right ovarian mass measured 12.2 cm x 8.5 cm x 6.9 cm. The cut surface revealed a multiloculated cyst containing hair strands and approximately 150 mL of greasy, yellowish fluid. A Rokitansky protuberance containing a tooth was identified.

Left ovarian mass measured 17.0 cm x 11.7 cm x 9.3 cm. The cut surface showed a unilocular cyst filled with approximately 200 mL of dark brown, hemorrhagic fluid. A Rokitansky nodule containing sebaceous material and hair was also present.

Microscopic examination

Right ovary sections demonstrated mature tissues derived from all three germ cell layers with no primitive elements. Ectodermal derivatives included stratified squamous epithelium, sebaceous glands, hair follicles, and neural tissue. Mesodermal components consisted of cartilage, smooth muscle, and mature adipose tissue. Endodermal elements were represented by intestinal type glandular structures. The final diagnosis was mature cystic teratoma.

Left ovary sections revealed extensive hemorrhagic necrosis, dilated congested blood vessels, and ghost outlines of mature teratomatous elements such as hair follicles and smooth muscle. No viable primitive cells were identified. The findings were consistent with hemorrhagic necrosis secondary to torsion of a mature cystic teratoma.

The combination of acute urinary retention, a palpable abdominal mass, and imaging findings led to the primary differential diagnoses of ovarian torsion complicating a neoplasm (benign vs. malignant germ cell tumor). Emergency surgery was indicated for both detorsion and definitive diagnosis.

Her postoperative course was managed with a 48-hour course of intravenous antibiotics for surgical prophylaxis (cefuroxime and metronidazole) and analgesia (paracetamol). She was subsequently transitioned to a five-day oral course of the same antibiotics and continued on oral paracetamol for pain control, supplemented with a two-week course of oral vitamin C. The urethral catheter was removed within 24 hours. Her post-operative packed cell volume on the second day was 30%. Her recovery was uneventful, and she was discharged home on the fourth post-operative day.

Histopathological analysis one month later revealed a right-sided mature cystic teratoma and left-sided hemorrhagic necrosis consistent with torsion. Peritoneal fluid cytology was negative for malignancy. The patient was commenced on daily hormone replacement therapy (Premarin) and placed on a three-monthly follow-up schedule with serial ultrasounds. At two-year follow-up, she remains stable with no complaints.

## Discussion

This case presents important lessons for clinical practice. First, acute urinary retention in a prepubertal girl should be considered a red flag for a significant pelvic mass until proven otherwise, which necessitates a thorough abdominopelvic examination and urgent imaging. Second, while imaging is essential, it must be interpreted with caution; radiologic features of complexity or suspicion for immaturity do not preclude a fertility-preserving surgical approach, as histopathology remains the diagnostic gold standard. Finally, the management of such cases must be multidisciplinary from the outset, involving pediatric surgeons, gynaecologists, endocrinologists, and radiologists, with a preoperative plan firmly centred on ovarian conservation even in the face of dramatic intraoperative findings like torsion and ischemia. This approach balances the acute surgical emergency with the patient's long-term endocrine and reproductive health.

Acute urinary retention has rare but serious complications in children with large pelvic masses. The most common pathophysiology process is the compression of the urinary bladder caused by the mass of the urinary tract. A recent study on ovarian teratomas in premenarchal girls had shown that abdominal pains were the most common symptoms of the condition (95 per cent), lower urinary tract symptoms like dysuria or frequency were reported by only 12 per cent, and actual urinary retention was extremely rare, reported in only a handful of case series [4]. The presented case echoes the need for thorough pelvic and abdominal examination with the use of advanced imaging techniques in young girls who have unexplained urinary retention despite a lack of canonical gynaecological symptoms. 

The pathophysiological quandary conveyed through the iteration of the two giant and morphologically intricate masses on each side is a primary learning event. A CT scan was done initially, with a possible diagnosis of bilateral immature ovarian teratoma notified of the possibility of malignancy. However, the later histopathological examination proved one mature cystic teratoma on the right side and hemorrhagic necrosis secondary to torsion on the left. The fact that the radiologic suspicion and definite pathology do not quite agree is not a miracle. A 2025 study by Mirfendereski and Mansouri argues that CT and MRI, as effective as they may be in identifying tissue contents and disease progression, have limited abilities to distinguish mature and immature teratomas, especially in cases where they are complicated by torsion and hemorrhage, which mimic malignancies [6]. The normal serum CA-125 level of the patient was encouraging, but the level of the diagnostic process is limited by the germ cell tumours, in which markers like AFP and β-hCG have proved to be more relevant. The normal AFP and β-hCG were reassuring against a secreting malignant germ cell tumor, aligning with the final benign histology, while the elevated LDH likely reflected tissue ischemia from torsion This finding is corroborated by the results of a recent multi-institutional study by Knaus et al. [7], which concluded that definitive diagnosis and determination of ovarian masses in children cannot rely solely on imaging but, in fact, on histopathology and findings at the time of operations [7]. 

The treatment plan highlights the changing quality of care in pediatric and adolescent gynaecology, especially fertility-saving surgery. Despite torsion, ischemia, and the size of the left ovarian mass (18x14cm), a cystectomy was performed successfully. The contemporary evidence confirms this approach. Bandhon et al. [8] stated that, despite a necrotic-looking ovary, detorsion and cystectomy must be attempted, as ovarian tissue has been proven to recover its functions robustly in most cases [8]. The literature supports a conservative surgical approach, as thromboembolic events are infrequent following detorsion and preservation of ovarian endocrine and reproductive function is of considerable importance. The present case, involving bilateral cystectomy with a successful outcome despite challenging intraoperative findings, exemplifies this principle. 

Lastly, postoperative discovery of a rudimentary uterus and the following initiation of hormone replacement therapy (HRT) brings in a critical aspect of long-term care. Although the acute ovarian pathology was the primary focus of the clinical attention, the uterine anomaly can indicate a possible underlying disorder of sex development (DSD) or a congenital anomaly of the Müllerian ducts. The necessity of HRT proves a loss of ovarian follicular reserve after bilateral surgery, which is a risk, although an effort is made to preserve the ovaries. In a cohort study, Tsui et al. emphasised the importance of monitoring pubertal development and skeletal density, as well as the timing of HRT initiation, to ensure normal growth and minimise the long-term consequences of skeletal fragility, such as osteoporosis [9]. The follow-up protocol of the patient, including the use of serial ultrasounds and cooperation with the endocrinology of children, agrees with these best-practice guidelines [10].

## Conclusions

This case provides a multifaceted learning opportunity. It reinforces that ovarian pathology can present dramatically in prepubertal children, that imaging must be interpreted with caution, and that a conservative, fertility-preserving surgical approach is the gold standard. Furthermore, it highlights the necessity of a long-term, multidisciplinary follow-up plan to address potential hormonal deficiencies and ensure holistic patient well-being into adulthood.
